# Supplementary material for: Multifunctional Characteristics of BCTH:0.5% Sm3+ Ceramics Prepared via Hydrothermal Method and Powder Injection Molding
Source: Materials (Basel). 2023 Oct 27;16(21):6910. doi: 10.3390/ma16216910 (PMC10650271; doi:10.3390/ma16216910)
Supplement: Supplementary file 1 [file materials-16-06910-s001.zip › materials-2666626-supplementary.pdf]

Supporting information for

Powder dependence of multifunctional characteristics of BCTH:0.5% Sm^{3+} ceramics prepared by hydrothermal method via powder injection molding

XRD data of reference standard materials used for Rietveld refinement in Figure 2a and 2b.

$BaTiO_3$ Amm2-PDF#81-2200

2-Theta	d(?)	I(f)	(h k l)	Theta	1/(2d)	2pi/d	n^2
22.104	4.0182	18.4	(0 1 1)	11.052	0.1244	1.5637	
22.277	3.9874	9.5	(1 0 0)	11.138	0.1254	1.5758	
31.417	2.8451	30.0	(0 0 2)	15.709	0.1757	2.2085	
31.585	2.8303	100.0	(1 1 1)	15.792	0.1767	2.2199	
38.853	2.3160	19.7	(1 0 2)	19.426	0.2159	2.7130	
38.924	2.3119	22.7	(1 2 0)	19.462	0.2163	2.7177	
45.088	2.0091	27.7	(0 2 2)	22.544	0.2489	3.1274	
45.456	1.9937	13.8	(2 0 0)	22.728	0.2508	3.1515	
50.706	1.7989	3.0	(0 1 3)	25.353	0.2779	3.4928	
50.848	1.7942	6.7	(0 3 1)	25.424	0.2787	3.5019	
51.100	1.7859	3.9	(2 1 1)	25.550	0.2800	3.5181	
56.037	1.6398	17.3	(1 1 3)	28.018	0.3049	3.8318	
56.144	1.6369	19.2	(1 3 1)	28.072	0.3055	3.8385	
56.299	1.6327	17.8	(2 0 2)	28.150	0.3062	3.8483	
65.570	1.4225	4.2	(0 0 4)	32.785	0.3515	4.4169	
65.765	1.4188	5.7	(0 4 0)	32.883	0.3524	4.4286	
65.954	1.4152	15.3	(2 2 2)	32.977	0.3533	4.4399	
70.187	1.3398	2.0	(1 0 4)	35.094	0.3732	4.6896	
70.443	1.3356	2.8	(1 4 0)	35.221	0.3744	4.7045	
70.537	1.3340	1.8	(2 3 1)	35.268	0.3748	4.7099	
74.562	1.2717	3.8	(0 2 4)	37.281	0.3932	4.9409	
74.699	1.2697	8.5	(1 3 3)	37.349	0.3938	4.9486	
75.239	1.2619	5.2	(3 1 1)	37.620	0.3962	4.9792	
78.956	1.2115	3.6	(1 2 4)	39.478	0.4127	5.1861	
79.092	1.2098	2.8	(1 4 2)	39.546	0.4133	5.1935	
79.533	1.2042	2.1	(3 0 2)	39.767	0.4152	5.2177	

$BaTiO_3$ R3m-PDF#86-1569

2-Theta	d(?)	I(f)	(h k l)	Theta	1/(2d)	2pi/d	n^2
22.183	4.0040	24.9	(0 1 0)	11.092	0.1249	1.5692	
31.538	2.8345	75.1	(1 1 0)	15.769	0.1764	2.2167	
31.611	2.8280	100.0	(-1 1 0)	15.805	0.1768	2.2217	
38.836	2.3170	9.1	(1 1 1)	19.418	0.2158	2.7118	
38.958	2.3100	24.8	(-1 1 1)	19.479	0.2165	2.7200	
45.257	2.0020	38.9	(0 2 0)	22.629	0.2498	3.1385	
50.907	1.7923	6.2	(1 2 0)	25.454	0.2790	3.5057	
51.006	1.7890	7.5	(-1 2 0)	25.503	0.2795	3.5121	
56.113	1.6377	11.3	(1 2 1)	28.056	0.3053	3.8366	
56.298	1.6328	21.3	(-1 2 1)	28.149	0.3062	3.8482	
65.846	1.4172	10.2	(2 2 0)	32.923	0.3528	4.4334	
66.014	1.4140	14.8	(-2 2 0)	33.007	0.3536	4.4435	
70.336	1.3374	1.3	(2 2 1)	35.168	0.3739	4.6982	
70.499	1.3347	2.8	(0 3 0)	35.249	0.3746	4.7077	
74.882	1.2670	8.5	(1 3 0)	37.441	0.3946	4.9590	
75.001	1.2653	9.0	(-1 3 0)	37.500	0.3952	4.9657	
79.156	1.2090	2.1	(1 3 1)	39.578	0.4136	5.1971	
79.391	1.2060	3.3	(-3 1 1)	39.695	0.4146	5.2099	

$BaTiO_3$ P4mm-PDF#89-1428

2-Theta	d(?)	I(f)	(h k l)	Theta	1/(2d)	2pi/d	n^2
22.169	4.0065	24.7	(0 0 1)	11.085	0.1248	1.5682	
31.512	2.8367	100.0	(1 0 1)	15.756	0.1763	2.2149	
38.867	2.3152	25.4	(1 1 1)	19.433	0.2160	2.7139	
45.103	2.0085	10.9	(0 0 2)	22.551	0.2489	3.1283	
45.227	2.0032	24.2	(2 0 0)	22.614	0.2496	3.1365	
50.809	1.7955	3.2	(1 0 2)	25.404	0.2785	3.4994	
50.923	1.7918	5.4	(2 0 1)	25.461	0.2791	3.5067	
56.163	1.6364	16.8	(1 1 2)	28.082	0.3056	3.8397	
65.787	1.4184	6.0	(2 0 2)	32.893	0.3525	4.4299	
65.884	1.4165	4.0	(2 2 0)	32.942	0.3530	4.4357	
70.354	1.3370	1.4	(2 1 2)	35.177	0.3740	4.6993	
74.680	1.2700	1.8	(1 0 3)	37.340	0.3937	4.9476	
74.863	1.2673	3.8	(3 0 1)	37.431	0.3945	4.9579	
79.031	1.2106	0.9	(1 1 3)	39.515	0.4130	5.1902	
79.211	1.2083	1.7	(3 1 1)	39.605	0.4138	5.2001	

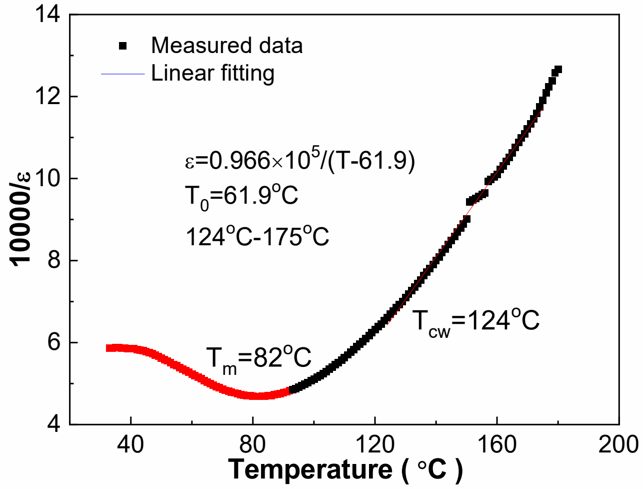

Figure S1. Dielectric response behavior fitted by Curie-Weiss law of BCTH:0.005Sm^{3+} ceramics prepared by hydrothermal method sintered under 1300 °C for 8 h using 10 kHz data.
